# Bismuth Oxide (Bi_2_O_3_) Nanoparticles Cause Selective Toxicity in a Human Endothelial (HUVE) Cell Line Compared to Epithelial Cells

**DOI:** 10.3390/toxics11040343

**Published:** 2023-04-04

**Authors:** Mohd Javed Akhtar, Maqusood Ahamed, Hisham Alhadlaq

**Affiliations:** 1 King Abdullah Institute for Nanotechnology, King Saud University, Riyadh 11451, Saudi Arabia; 2 Department of Physics and Astronomy, College of Sciences, King Saud University, Riyadh 11451, Saudi Arabia

**Keywords:** endothelial cells, oxidative stress, anti-angiogenic potential, cancer therapy

## Abstract

A review of recent literature suggests that bismuth oxide (Bi_2_O_3_, referred to as B in this article) nanoparticles (NPs) elicit an appreciable response only after a concentration above 40–50 µg/mL in different cells all having an epithelial origin, to the best of our knowledge. Here, we report the toxicological profile of Bi_2_O_3_ NPs (or BNPs) (71 ± 20 nm) in a human endothelial cell (HUVE cell line) in which BNPs exerted much steeper cytotoxicity. In contrast to a high concentration of BNPs (40–50 µg/mL) required to stimulate an appreciable toxicity in epithelial cells, BNPs induced 50% cytotoxicity in HUVE cells at a very low concentration (6.7 µg/mL) when treated for 24 h. BNPs induced reactive oxygen species (ROS), lipid peroxidation (LPO), and depletion of the intracellular antioxidant glutathione (GSH). BNPs also induced nitric oxide (NO,) which can result in the formation of more harmful species in a fast reaction that occurs with superoxide (O_2_^•−^). Exogenously applied antioxidants revealed that NAC (intracellular GSH precursor) was more effective than Tiron (a preferential scavenger of mitochondrial O_2_^•−^) in preventing the toxicity, indicating ROS production is extra-mitochondrial. Mitochondrial membrane potential (MMP) loss mediated by BNPs was significantly less than that of exogenously applied oxidant H_2_O_2_, and MMP loss was not as intensely reduced by either of the antioxidants (NAC and Tiron), again suggesting BNP-mediated toxicity in HUVE cells is extra-mitochondrial. When we compared the inhibitory capacities of the two antioxidants on different parameters of this study, ROS, LPO, and GSH were among the strongly inhibited biomarkers, whereas MMP and NO were the least inhibited group. This study warrants further research regarding BNPs, which may have promising potential in cancer therapy, especially via angiogenesis modulation.

## 1. Introduction

Nanoparticles (NPs) of bismuth (Bi) chalcogenides, which include bismuth oxide, bismuth sulfide, bismuth selenide, and bismuth telluride, have received significant attention because of their potential therapeutic purposes [1]. Bismuth (Bi) has traditionally been used as an active component in many pharmacological products. Nanoparticles of bismuth oxide (Bi_2_O_3_ NPs or BNPs) have exhibited strong potential in the field of antimicrobial formulations, combined cancer therapy, bioimaging, and tissue engineering, and are regarded as a breakthrough in biomedical applications [1]. Their expansion in NP form is a recent development that can yield a potential improvement in the field of antimicrobial formulations, combined cancer therapy, bioimaging, and tissue engineering [1,2]. In addition to radiosensitizers, BNPs per se have been reported as having antimicrobial activity, as well as being an inducer of differential toxicities in normal and cancer cell lines [3,4]. BNPs have also been reported to have positive synergistic potential for chemo- and radio-therapy in MCF-7 and MDA-MB-231 breast cancer cell lines [5].

As a result of their recent introduction, emerging toxicologic data on BNPs are inadequate and, therefore, also inconsistent. Needless to say, the sensitivity and, therefore, toxicity of BNPs vary with different cell types that reflect the diversity of cell phenotypes. A review of the effective concentration of BNPs carried out by several investigators [6,7,8,9,10,11] suggests that a concentration range around 40–100 µg/mL can result in significant inhibition of cell growth [6,7,8,9,10,11]. However, all of the above-mentioned biological activity attributed to BNPs is carried out in cells of epithelial origin, but not in endothelial cells, to the best of our knowledge. We were, therefore, prompted to carry out a bio-response analysis of BNPs in endothelial-type cells, as endothelial cell mediated angiogenesis modulation is at the core of mechanisms responsible for metastasis [12]. Any agent that may selectively inhibit endothelial cells could be of significant value for advancing studies based on angiogenesis modulation for cancer therapy. Endothelial cells are a recommended cell type in advancing the mechanism of cancer therapy, as agents toxic to endothelial cells can be potentially anti-angiogenic [12].

In our attempt at screening a NP for its selective inhibitory effect on endothelial cells, we surprisingly observed that a much lower concentration of BNPs resulted in cytotoxicity in a human endothelial cell line (HUVE cells), as explained later in this study. Nitric oxide (NO) and O_2_^•−^ radicals have a decisive role in the functioning of endothelial cells in physiological and pathological conditions. Therefore, we assessed NO and O_2_^•−^ levels in control and BNP-treated HUVE cells by applying direct and indirect methods of investigation, followed by other relevant markers of oxidative stress such as lipid peroxidation and mitochondrial function, as shown in some recent publications [13,14,15]. LPO was assessed using live cell imaging by applying BODIPY dye, and using the biochemical method of quantifying thiobarbituric acid reactive substances (TBARS). Ratiometric dye JC-1 was applied to detect potential changes in mitochondrial membrane potential (MMP) in HUVE cells. Exogenous antioxidants were further employed to understand the preferential component of ROS, if any, involved in the mechanism of toxicity in HUVE cells brought about by BNP interaction. These antioxidants include N-acetyl-L-cysteine (NAC), which functions as a GSH precursor across cell cytoplasm [16], and Tiron (sodium 4,5-dihydroxybenzene-1,3-disulfonate) molecules, which function as ROS scavengers preferentially in mitochondria [17]. General antioxidant NAC and mitochondria-specific Tiron antioxidant can also provide in-depth information about the involvement of mitochondria and the site of ROS production.

## 2. Materials and Methods

### 2.1. Chemicals and Reagents

Fetal bovine serum, calcein-AM, BODIPY-red, and penicillin-streptomycin were purchased from Invitrogen Co. (Carlsbad, CA, USA). DMEM F-12, MTT [3-(4,5-dimethyl thiazol-2-yl)-2,5-diphenyl tetrazolium bromide], NADH, pyruvic acid, perchloric acid, DHE, DCFH-DA, DAR-1 (4,5-Diamino-N,N,N′,N′-tetraethylrhodamine), JC-1, GSH, o-phthalaldehyde (OPT), Hank’s balanced salt solution (HBSS), caspase substrates, and Bradford reagent were obtained from a commercial source (Sigma–Aldrich, St. Louis, MO, USA). Ultrapure water was taken from a Milli-Q system (Millipore, Bedford, MA, USA). All other chemicals used were of reagent grade.

### 2.2. Electron Microscopy

Methods for the synthesis and physico-chemical characterization of BNPs have been published in several reports of our colleagues [6,9], as have been the methodologies for transmission electron microscopy (TEM) and scanning electron microscopy (SEM).

### 2.3. Cell Culture and Treatment with BNPs

HUVE, A549, MCF-7, and HepG2 cells (ATCC, US) were all cultured in a DMEM medium that was supplemented with extra endothelial growth factors and chemicals (CADMEC, Cell Applications, Inc., San Diego, CA, USA). Additionally, culture medium was complimented with 10% fetal bovine serum, 100 U/mL penicillin, and 100 µg/mL streptomycin. Cells were incubated at 37 °C in a humidified 5% CO_2_ incubator and passaged for every interval of 2–3 days.

### 2.4. Determination of Cell Viability by MTT

Cell viability in the two cell types was determined by a biochemical measurement of oxidoreductase enzymes in viable cells. The reductive potential of these enzymes in viable cells was evaluated by the conversion of the yellow MTT probe to reduced blue-colored formazan crystals [18]. Later, formazan crystals were solubilized in an aqueous solution of 20% SDS dissolved in 50% dimethylformamide. Briefly, 2 × 10^4^ cells were seeded in each well of a 96-well plate and treated the next day. After a 24 h exposure period, cells were added with filtered MTT solution made in HBSS and left for 2.5 h. Formazan crystal thus formed by viable cells was solubilized in 20% SDS prepared in 50% dimethylformamide. Absorbance at 570 nm was determined in a plate reader (Synergy HT, Bio-Tek, Winooski, VT, USA) and cell viability was calculated as % of control. IC50s calculations for the NPs and H_2_O_2_ were undertaken using the online IC50 calculator (https://www.aatbio.com/tools/ic50-calculator) (accessed on 18 February 2023) provided by AAT Bioquest, Inc. (Pleasanton, CA, USA). Cells were also imaged using phase contrast microscopy.

### 2.5. Detection of Intracellular ROS

The potential induction of ROS was determined by a 2′, 7′-dichlorofluorescin diacetate (DCFH-DA) probe [19] which was incubated for 45 min at the final concentration of 50 µM. After the treatment period was over, cells were washed twice with cold PBS, removing excess dye, and the resultant DCF fluorescence was measured at 528 nm in a plate reader (Synergy HT, Bio-Tek, Winooski, VT, USA).

### 2.6. Measurement of Intracellular GSH

The cellular content of GSH was quantified according to the method given by Hissin and Hilf [20]. Control and treated cells were collected and lysed in an aqueous solution of 0.1% deoxycholic acid plus 0.1% sucrose and centrifuged at 10,000× *g* for 10 min at 4 °C. Supernatant was precipitated in 1% perchloric acid and centrifuged again at 10,000× *g* for 5 min at 4 °C to obtain protein-free supernatant. A quantity of 20 μL from the protein-precipitated sample was mixed with 160 μL of GSH assay buffer (0.1 M phosphate–5 mM EDTA, pH 8.3) and 20 μL of thiol-reactive o-phthalaldehyde (OPT, 1 mg/mL in methanol). After 2.5 h of incubation at room temperature in the dark, fluorescence was measured at an emission wavelength of 460 nm (Synergy HT, Bio-Tek, Winooski, VT, USA), along with similarly prepared standards of GSH. Protein was estimated from unprecipitated supernatant and data were converted to GSH nmol/mg protein.

### 2.7. Analysis of Intracellular NO

Intracellular NO was quantified by a rhodamine-based live-cell-permeable fluorescent probe, DAR-1, which reacts specifically with intracellular NO, generating an intense fluorescence in the infra-red region [21,22,23]. Cells in a 12-well plate were labeled with DAR-1 at a final concentration of 15 μM for 2 h. Cells were also co-labeled with a live cell fluorescent probe calcein-AM at 1 μM, corroborating the live cell status. Then, cells were carefully washed with cold HBSS three times and imaging was conducted using an appropriate filter in a microscope (Leica DMi8, Wetzlar, Germany).

### 2.8. Analysis of Membrane Damage

The intactness of the cell membrane was determined by biochemical methods, followed by direct observation of live cells under a suitable fluorescent probe. Lactate dehydrogenase (LDH) release was measured by taking 100 µL centrifuged culture media and mixing it in a total volume of 3.0 mL of LDH assay cocktail (100 μL of 6 mM Na-pyruvate, 100 μL of 0.4 mM NADH, and 2.7 mL 0.1 M K-phosphate buffer, pH 7.4) [24], and NADH absorbance at 340 nm was recorded at 25 °C using a spectrophotometer (Genesys 10 Bio, Thermo Fisher Scientific, Madison, WI, USA). The basic mechanism of membrane damage caused by lipid peroxidation in fatty acids was determined by quantifying TBARS (thiobarbituric acid reactive substances) using the method of Ohkawa et al. [25]. Membrane integrity was confirmed by direct observation of cells labeled with a lipophilic C11-BODIPY581/591 probe (Life Technologies-Invitrogen, Carlsbad, CA, USA) as described elsewhere [26,27]. This is a ratio metric dye that is incorporated in membrane bilayers of cells and emits uniform red fluorescence in a non-oxidized state, and brighter green fluorescence in an oxidized state which is proportional to the amount of ROS [28]. Briefly, cells in a 12-well culture plate were labeled with a freshly prepared C11-BODIPY581/591 probe in HBSS at the final concentration of 2 μM and incubated for 60 min in the dark. Cells were carefully washed three times with HBSS, removing excess dye, and imaged under a fluorescence microscope (Leica DMi8 manual, Wetzlar, Germany).

### 2.9. Determination of Mitochondrial Membrane Potential by JC-1

Mitochondrial membrane potential (MMP) in control cells and cells treated with NPs was determined by JC-1. In healthy cells, JC-1 spontaneously forms complexes known as J-aggregates with intense and discrete red fluorescence. In apoptotic or unhealthy cells, JC-1 remains in the monomeric form, which emits bright but diffuse green fluorescence [29]. When the treatment period was over, media were aspirated from each well and labeled with 5 µM JC-1 in Hepes-buffered HBSS for 20 min. Fluorescence pictures of JC-1 monomer and aggregate in cells were captured using a fluorescence microscope (Leica DMi8, Germany). Colocalization of the two states of JC-1 was demonstrated by merging the two images in ImageJ software.

### 2.10. Protein Estimation

The total protein content was measured by a convenient BCA Protein Assay Kit from Sigma–Aldrich, St. Louis, MO, USA as per instructions.

### 2.11. Statistics

ANOVA (one-way analysis of variance) followed by Dunnett’s multiple comparison tests were employed for statistical analysis of results. For a particular set of experiments, a burst of images was captured at the constant exposure of time, gain, saturation, and gamma. Corrected total cellular fluorescence (CTCF) was calculated by subtracting background fluorescence (without the cell) from the mean of cellular fluorescence using ImageJ software (NIH, Bethesda, MD, USA).

## 3. Results

### 3.1. BNPs Killed HUVE Cells at Significantly Lower Concentrations Compared to Cells with Epithelial Origin

**Figure 1:**
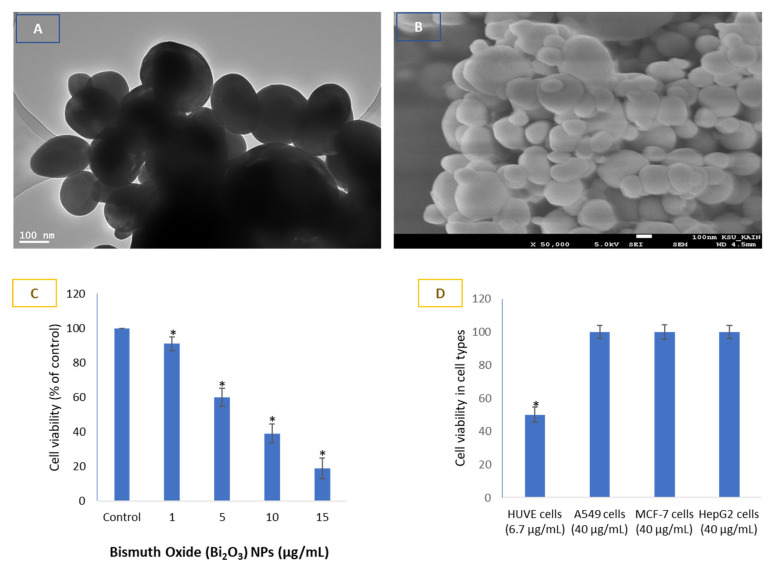
Shapes and sizes of bismuth oxide nanoparticles (BNPs) (70 nm) and their bio-response in endothelial cells with EC50 of 6.7 μg/mL exposed to 24 h. BNPs appear spherical in shape as evidenced by images captured by transmission electron microscopy (**A**) and scanning electron microscopy (**B**). Bar length in (**A**,**B**) corresponds to 100 nm. Concentration-dependent cell viability in HUVE cells (**C**). Cytotoxicity induced by BNPs in HUVE cells was compared with the cytotoxicity potential in cells of epithelial origins (**D**). Data suggest BNPs to be potently toxic in endothelial cells with an EC50 of 6.7 μg/mL while exhibiting little sign of toxicity in epithelial cells exposed to up to 40 μg/mL for 24 h. The epithelial cells were chosen because of their easy availability in our lab. The numerical value in ‘μg’ given for each cell type represents the concentration in μg/mL. EC50, defined as the concentration of the substance at which the response is half of its maximal response, was calculated using an online calculator (https://www.aatbio.com/tools/ic50-calculator) (accessed on 18 February 2023) provided by AAT Bioquest, Inc. (San Francisco, CA 94085, USA) using a ‘three parameter mode’. Data represented are mean ± SD of three identical experiments (*n* = 3) conducted in triplicate. * Statistically significant difference compared to the controls (*p* < 0.05).

For the sake of information purposes, the average size of BNPs was calculated to be 71 ± 20 nm, as synthesized, characterized and reported previously by our research colleagues [6,9,11]. We provide, however, unpublished TEM and SEM image of BNPs in Figure 1A,B, respectively. Hence, we would like to present our main findings related to the interaction of BNPs with HUVE cells only observed in this study. The IC50 of BNPs in HUVE cells was calculated to be 6.7 µg/mL when exposed for 24 h (Figure 1C). Contrary to the toxicity in HUVE cells, no appreciable toxicity of BNPs was found when exposed to a concentration up to 40 µg/mL for 24 h in epithelial cells of A549, HepG2, and MCF-7 (Figure 1D). Under similar conditions of BNP treatment, however, the effective concentration was much reduced in human umbilical vein-derived endothelial (HUVE) cells compared to those of the non-responsive concentration of BNPs tested (i.e., 40 µg/mL) in epithelial cells, as stated in the introduction (Figure 1A,B).

### 3.2. BNPs Caused Significant O_2_^•−^ Generation in HUVE Cells

**Figure 2:**
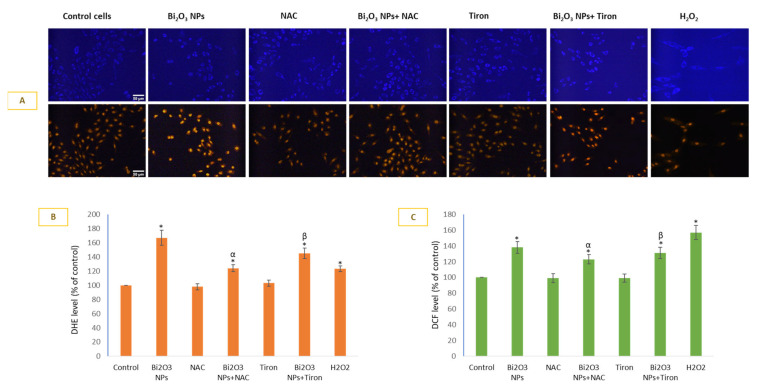
Production of O_2_^•−^ was measured by DHE live imaging (**A**) and DHE fluorescence calculation (**B**) in ImageJ software. DCFHDA was used to detect H_2_O_2_ (**C**). Data represented are mean ± SD of three identical experiments (*n* = 3) conducted in triplicate. * Statistically significant difference compared to the controls (*p* < 0.05). Any significant (*p* < 0.05) difference in response from that mediated by NPs and the addition of either NAC or Tiron is denoted by α and β, respectively.

Cell permeable dyes hydroethidine (DHE) and DCFH-DA were used to detect O_2_^•−^ (Figure 2A,B) and H_2_O_2_ (Figure 2C) production inside cells. Live cell DHE imaging (A) was conducted followed by respective CTCF calculations in individual cells (B). BNPs induced 1.67-fold O_2_^•−^ generation in HUVE cells in comparison to untreated control cells. In terms of effectiveness, NAC was more effective in inhibiting NPs-mediated O_2_^•−^ production in comparison to Tiron; O_2_^•−^ generation due to NP treatment was inhibited by 26% by NAC and 14% by Tiron. The trend for DCF fluorescence was similar, and is largely a function of H_2_O_2_ (see Figure 2C). In the case of exogenous treatment of H_2_O_2_, however, DHE fluorescence was 1.23-fold compared to that in the control, whereas DCF was 1.57-fold that in the control. In other words, exogenous H_2_O_2_ caused induction of DCF fluorescence (i.e., 1.57-fold the control), which was significantly higher in comparison to the NP-mediated induction (1.38-fold the control) of DCF fluorescence. In the case of DHE fluorescence, however, it was the NP that caused significant induction of DHE fluorescence (1.67-fold the control) in comparison to that of H_2_O_2_-mediated induction of DHE fluorescence (1.23-fold the control).

### 3.3. BNPs Caused Significant Lipid Peroxidation in Membranes of HUVE Cells

**Figure 3:**
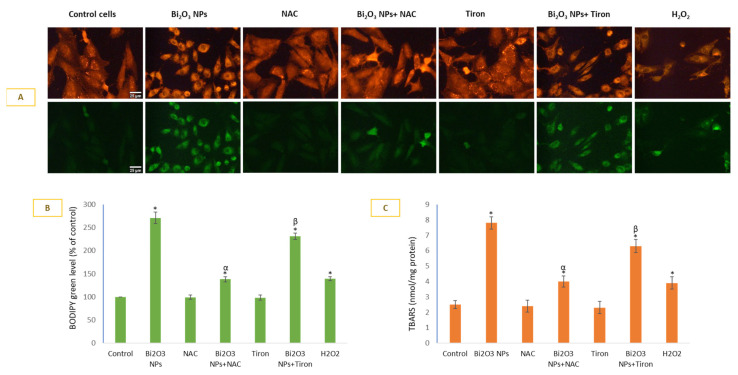
Lipid peroxidation (LPO) was detected using live-cell imaging dye (**A**) that preferentially localizes in a hydrophobic environment and fluoresces green in proportion to LPO. BODIPY green fluorescence was calculated in ImageJ software (**B**). LPO was also assessed by a more conventional method by quantifying TBARS spectrophotometrically (**C**). Data represented are mean ± SD of three identical experiments (*n* = 3) conducted in triplicates. * Statistically significant difference compared to the controls (*p* < 0.05). Any significant (*p* < 0.05) difference in response from that mediated by NPs and the addition of either NAC or Tiron is denoted by α and β, respectively.

BNPs caused significant lipid peroxidation in membranes of HUVE cells. Lipid peroxidation as measured by live cell imaging labeled with lipophilic probe BODIPY (Figure 3A BODIPY images and 3B BODIPY green fluorescence) fluorescence. BNP treatment caused 2.71-fold induction of LPO compared to the control when determined by the BODIPY method. A 2.7-fold BODIPY green fluorescence induction by NP was reduced to 1.38-fold by NAC and 2.31-fold by Tiron. Similarly, a 7.8-fold TBARS induction (3C) by NP was reduced to 4-fold by NAC and 6.3-fold by Tiron.

### 3.4. BNPs Significantly Induced NO Level That Was Least Preventable by Antioxidants in HUVE Cells

**Figure 4:**
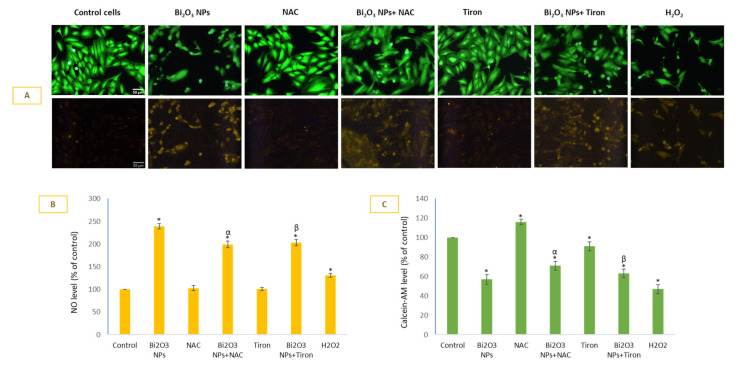
Generation of NO due to BNPs in HUVE cells was determined using NO-specific fluorescent probe DAR-2 (infra-red images in (**A**) and fluorescence plotting in (**B**) for individual cells minus non-cellular background). Note that NO generation was a cytotoxic event in HUVE cells due to BNPs exposure as NO fluorescence was in an inverse relationship with live cell dye fluorescence of calcein-AM (green images in A and fluorescence plotting in (**C**) for individual cells minus non-cellular background). Data represented are mean ± SD of three identical experiments (*n* = 3) conducted in triplicate. * Statistically significant difference as compared to the controls (*p* < 0.05). Any significant (*p* < 0.05) difference in response from that mediated by NPs and the addition of either NAC or Tiron is denoted by α and β, respectively.

BNPs induced significant NO production in HUVE cells (Figure 4A infra-red images in lower panel), and it was comparatively little affected by the two antioxidants, when compared to other biomarkers evaluated in this study. NAC and Tiron almost equally inhibited NO production due to NPs. The inhibitory effects of NAC and Tiron on NP-mediated NO production were 17 ± 3% and 15 ± 3% respectively. As can be seen in DAR-1 fluorescence quantification (Figure 4B), NO generation was inversely proportional to the live-cell dye calcein-AM (Figure 4A green images in upper panel, and 4 D calcein-AM-fluorescence plotting) suggesting NO generation due to BNPs as a cytotoxic event in HUVE cells. Recall that the higher the calcein-AM fluorescence in cells, the lower the cytotoxicity.

### 3.5. BNPs Caused Significant Loss in MMP and Depletion in Intracellular GSH

**Figure 5:**
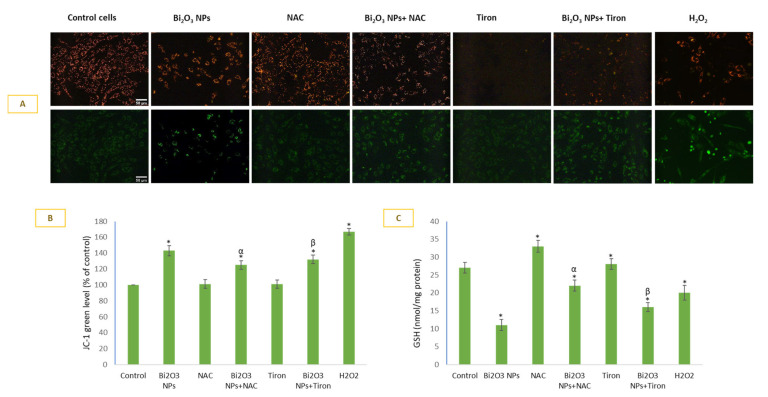
MMP was detected by JC-1 imaging in cells (**A**) and plotting green JC-1 fluorescence (**B**) in which green fluorescence is directly proportional to loss of MMP. Antioxidant GSH was also evaluated under various treatment conditions (**C**). Data represented are mean ± SD of three identical experiments (*n* = 3) conducted in triplicate. * Statistically significant difference compared to the controls (*p* < 0.05). Any significant (*p* < 0.05) difference in response from that mediated by NPs and the addition of either NAC or Tiron is denoted by α and β, respectively.

BNPs induced a loss of MMP in HUVE cells (Figure 5A, JC-1 cell images and Figure 5B, JC-1 green fluorescence plotting). IC50 of BNPs caused a 143% loss of MMP as compared to control cells. However, MMP loss due to NP exposure was significantly protected by the two antioxidants used in this study. A 1.43-fold increase in JC-1 green fluorescence caused by NP (in comparison to the control) was reduced to 1.25-fold (or 87% in comparison to the control) by NAC and 1.28-fold (or 90% in comparison to the control) by Tiron. Alternatively, MMP loss due to NP treatments was restored by 13% by NAC and 10% by Tiron. Interestingly, exogenously applied H_2_O_2_ caused more MMP loss (167% vs. the control) than due to BNP treatment (143% vs. control). BNPs treatment caused 2.4-fold exhaustion of GSH (Figure 5C) in comparison to the control. Exogenous H_2_O_2_ caused a 1.3-fold GSH depletion versus the control. NAC significantly restored GSH, and this could be another explanation for the NAC-mediated prevention of cells against BNPs-induced toxicity.

## 4. Discussion

The IC50 of BNPs in HUVE cells was calculated to be 6.7 µg/mL for a 24 h exposure time. The IC50s of BNPs in A549 cell, HepG2 cell, and primary rat hepatocyte were reported to be 205, 203, and 553 µg/mL, respectively, for 24 h exposure in a recent publication [11]. Similarly, significant toxicity of BNPs was noted in A549 and nasopharyngeal carcinoma (KB) only after a 50 µg/mL exposure for 24 h in another study [7]. Internalization of BNPs in many cancer cells has been reported by several investigators [7,30,31]. Over 40 µg/mL of BNPs (100 nm) was reported to induce cytotoxicity in MCF-7 cells, resulting in cell viability inhibition to 50% when exposed for 24 and 48 h [8]. Moreover, BNPs induced a significant amount of oxidative stress, mitochondrial dysfunction, and, consequently, apoptosis-like mode of cell death in MCF-7 cells [8]. In recent work, a 51% cell viability in human breast cancer (MCF-7) cells was reported at the concentration of 200 µg/mL of BNPs (97 nm) when exposed for 24 h [6]. Another study reported over 50 µg/mL of BNPs was capable of inducing significant toxicity in A549 cells and nasopharyngeal carcinoma (KB) cells when incubated for 24 h [7]. In line with the above findings, we also did not find any appreciable toxicity of BNPs in epithelial cells of A549, hepG2, and MCF-7 up to a concentration of 45 µg/mL when exposed for 24 h. Contrary to findings in epithelial cells, under similar conditions of treatment, BNPs induced potent cytotoxicity in the human endothelial HUVE cell line. The IC50 of BNPs in HUVE cells was calculated to be 6.8 µg/mL for 24 h exposure, which is much steeper compared to that in epithelial cells.

In NP-induced cytotoxicity, ROS are the key mediators of toxicity. Recall that ROS can have complex effects on the life of a cell. Depending on the magnitude and site of generation, ROS can induce cell proliferation by activating pro-survival factors, as well as causing cell deaths if the damage is beyond repair by commencing various modes of death program [32,33]. From the data observed in this study, it is safe to conclude that NPs cause toxicity in HUVE cells by inducing O_2_^•−^ rather than H_2_O_2_ as a major component of ROS. O_2_^•−^ could have later been converted to H_2_O_2_ by various superoxide dismutases (SODs). Depending on reaction environments, O_2_^•−^ can behave as both an oxidant and a reductant [34]. Especially under an acidic medium (i.e., in the presence of H^+^), O_2_^•−^ can be a powerful one-electron oxidant. In this study, we found high levels of O_2_^•−^and NO. We also observed strong lipid peroxidation that might have been initiated by O_2_^•−^ itself, as well as by radical species (i.e., ^•^NO_2_ and ^•^OH) derived from a fast reaction between O_2_^•−^ and NO leading to the unstable product ONOO^-^ [34]. For the sake of easier comparison, NAC inhibited NP-induced BODIPY fluorescence by 49% and NP-induced TBARS absorbance by 50%, whereas Tiron inhibited NP-induced BODIPY fluorescence by only 15% and TBARS absorbance by 19%. Of the two antioxidants used, NAC clearly exhibited the most powerful inhibitory effect on the NP-mediated LPO. NAC has been reported to inhibit LPO promoted by a wide variety of inorganic NPs [35,36]. Tiron cytoprotective potential, although less effective than NAC, also suggests the involvement of O_2_^•−^. Since NP-induced O_2_^•−^ production inhibition was greater under NAC than Tiron, the site of O_2_^•−^ production may be extra-mitochondrial (as discussed later, together with data on the smaller loss in MMP caused by NP than by exogenous H_2_O_2_). ROS can have dramatic effects in terms of biological outcomes, which may be site- and amount-dependent in a variety of cells [32,33]. Among the agents that generate extra-mitochondrial ROS, membrane NADPH oxidases constitute one of the major contributors, particularly under conditions of cell exposure to foreign agents such as bacteria, viruses, and ultra-fine particles [37].

NO is generated at physiological levels (in nanomolar quantities) that are required for proper signaling functions in various types of cells by constitutively active NO syntheses such as endothelial NOS (eNOS) and neuronal NOS (nNOS) [34]. Alternatively, inducible NOS (iNOS) can be activated in response to shear pressure, inflammation, and oxidative stress, which can lead to NO generation in micro-molar quantities within cells [34]. Overproduction of NO can lead to nitrosylation and nitrosation reactions with other cellular molecules present in cells, resulting in ‘nitrosative’ stress like ‘oxidative’ stress [38]. Moreover, the fast reaction between O_2_^•−^ and NO produces peroxynitrite (ONOO^-^), which is more deleterious than either precursor. ONOO^-^ is a member of reactive nitrogen species (RNS) [34]. The protonated form of ONOO^−^ (i.e., ONOOH, peroxynitrous acid) is more diffusible due to its neutral charge and stability [38]. Protonation is promoted under hydrophobic environments of membranes [34]. ONOOH is unstable and undergoes direct homolytic decomposition, resulting in strong oxidizing radical species (RNS and ROS) such as ^•^NO_2_ and ^•^OH, which can lead to lipid peroxidation in unsaturated fatty acid molecules [38]. Alternatively, NO can also terminate lipid peroxidation acting as an antioxidant in a context-dependent manner [38]. In essence, ROS and NO can have opposing effects in a context-dependent manner, and modulating ROS- and NO-dependent activity, as well as understanding angiogenesis and onco-biology, is an intense area of research in the field of cancer therapy. In this study, we found a significant induction of NO measured under live-cell imaging by cell-permeable NO-specific fluorescent DAR-2 [21]. BNPs induced significant NO production in HUVE cells. As can be seen in imaging data, NO generation was inversely proportional to the live-cell dye calcein-AM fluorescence, suggesting NO generation due to BNPs is a cytotoxic event in HUVE cells. Moreover, NO production was comparatively little affected by the two antioxidants, when compared to other biomarkers evaluated in this study, suggesting NO induction due to BNPs in HUVE cells should be further explored in future research. Like NO, which was affected little by the two antioxidants, NP-mediated loss in MMP was also little affected by the two antioxidants used in this study. BNPs caused a significant loss in MMP compared to the control; however, it was less than that caused by exogenously applied oxidant H_2_O_2_.

Altogether, BNPs caused greater induction on the level of LPO, O_2_^•−^, and NO in comparison to standard oxidant H_2_O_2_. BNPs also caused strong depletion of the antioxidant GSH. GSH is a powerful reducing agent as it can reduce disulfides of key proteins and enzymes to thiols, restoring their native functionality [39]. Disulfide can be induced in proteins by various oxidants that attack thiols (-SHs) of active site cysteine (Cys) residues, rendering them inactive or even dysfunctional [39]. Restoring GSH depletion and inhibiting cell membrane lipid peroxidation by NAC might be the key mechanism in the prevention of the potent toxicity induced by BNPs in HUVE cells. The next piece of information regarding the site of O_2_^•−^ production due to NPs emerges from the use of NAC and mitochondria preferring Tiron. Tiron also showed significantly less inhibition of LPO compared to NAC. Since the mitochondria-preferring O_2_^•−^ scavenger antioxidant Tiron was less effective than the GSH precursor NAC, NP-induced O_2_^•−^ production might be extra-mitochondrial. Another piece of evidence is the data on MMP in which MMP loss mediated by NP was significantly less than that of exogenously applied oxidant H_2_O_2_. In addition, MMP loss was not as strongly inhibited by the two antioxidants as the inhibitory effect found for markers O_2_^•−^, LPO, and intracellular GSH. When we compared the inhibitory capacities of the two antioxidants on different parameters of this study, ROS, LPO, and GSH were among the strongly inhibited biomarkers, whereas MMP and NO were the least inhibited group.

## 5. Conclusions

As inferred from DHE and DCF data, NP toxicity seems to occur through the production of O_2_^•−^ rather than H_2_O_2_. Membrane peroxidation data reflect that NPs can have direct interaction with cells, resulting in strong peroxidation-mediated toxicity. LPO induction and GSH depletion are strongly inhibited by NAC, but not by Tiron. Production of NO due to BNPs in HUVE cells is interesting because NAC and Tiron have very little effect on NO induction, suggesting further exploration is warranted with an emphasis on detailed NO-biology in medicine. This study suggests that BNPs preferentially target HUVE cells. Endothelial-cell-dependent angiogenesis plays a crucial role in cancer cell dissemination from the original tissue to distant tissue during cancer metastasis/malignancy. This property of preferential killing of HUVE cells by BNPs makes them a potential candidate for advancing research to examine the potential effect of BNPs on the angiogenesis modulation.

## Data Availability

Data is contained within the article.
